# Supplementary material for: The Effects of Acid and Water in the Formation of Anodic Alumina: DFT and Experiment Study
Source: Molecules. 2023 Mar 7;28(6):2427. doi: 10.3390/molecules28062427 (PMC10052643; doi:10.3390/molecules28062427)
Supplement: Supplementary file 1 [file molecules-28-02427-s001.zip › molecules-2207820-supplementary.pdf]

Supplementary Materials

The Effects of Acid and Water in the Formation of Anodic Alumina: DFT and Experiment Study

Zhengwei Zhang [1,*], Jin Kang [1], Xiaodong Li [1], Ping Li [1], Yali Du [1], Yufan Qin [1], Ningyi Li [1] and Jiebin Li [2]

[1] High Value Fine Chemicals Research Center, Department of Chemistry & Chemical Engineering, Jinzhong University, Jinzhong 030619, China
[2] Shaanxi Applied Physics and Chemistry Research Institute, Xi'an 710061, China
* Correspondence: zhangzhw1225@163.com

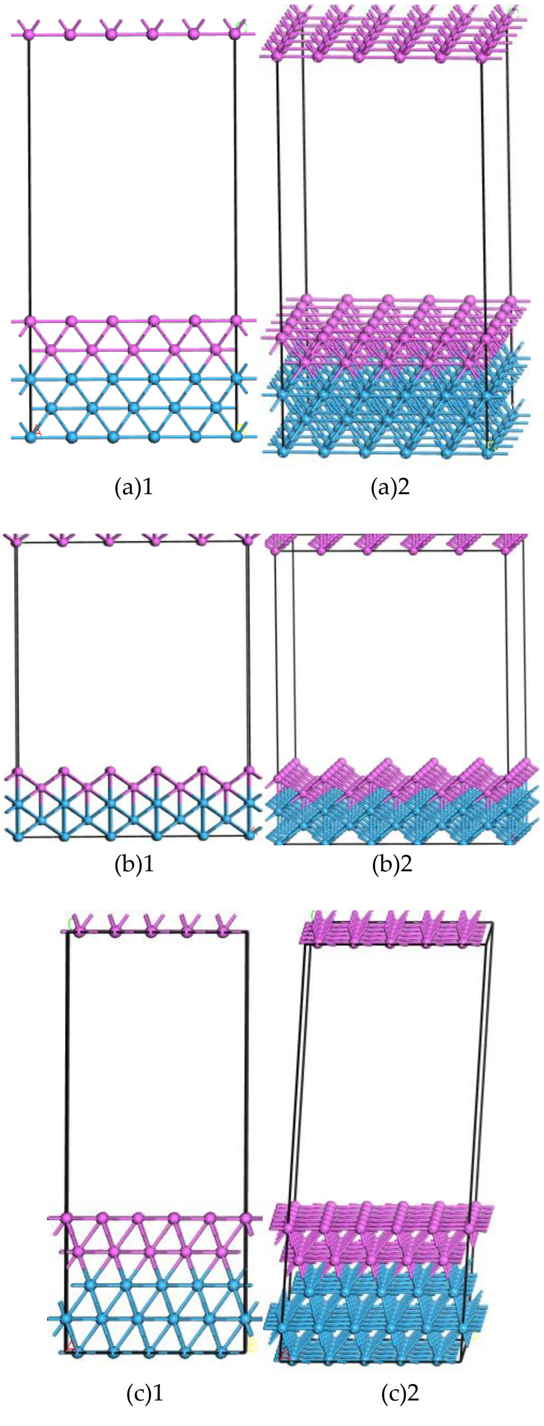

Figure S1. The aluminium slabs of three faces. The coordinates of the blue atoms are fixed, other atoms relax freely.
(a)1: the side view of (100) slab; (a)2: the oblique view of (100) slab;
(b)1: the side view of (110) slab; (b)2: the oblique view of (110) slab;
(c)1: the side view of (111) slab; (c)2: the oblique view of (111) slab.

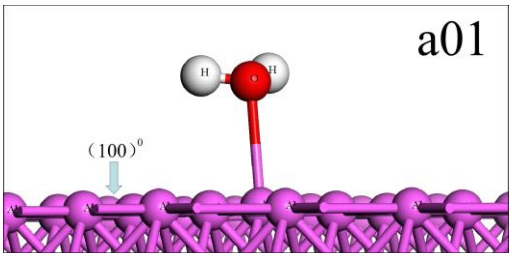

(a)01

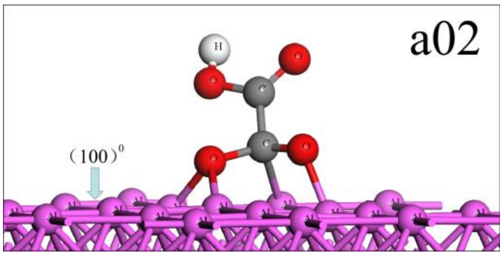

(a)02

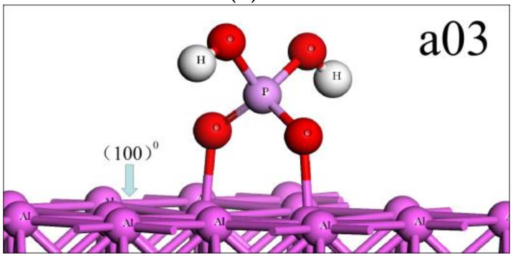

(a)03

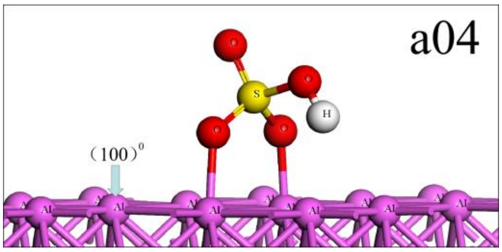

(a)04

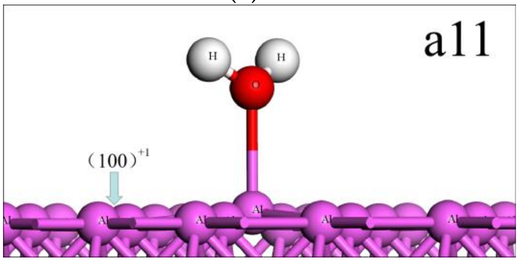

(a)11

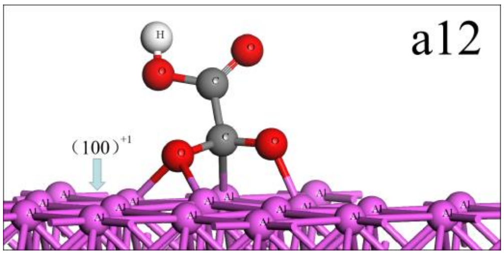

(a)12

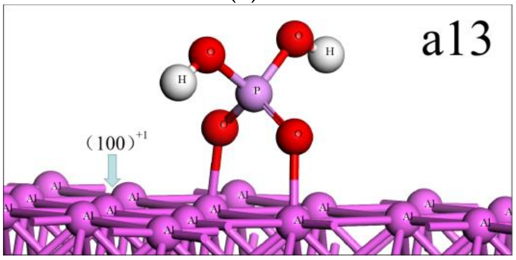

(a)13

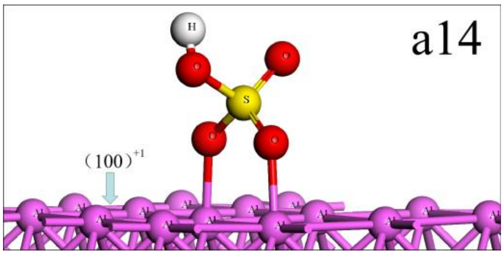

(a)14

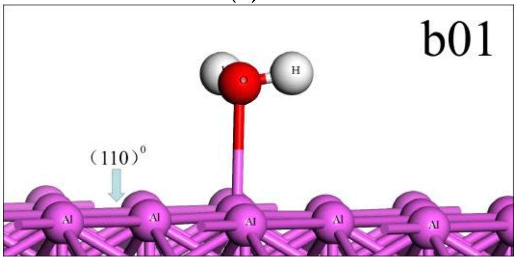

(b)01

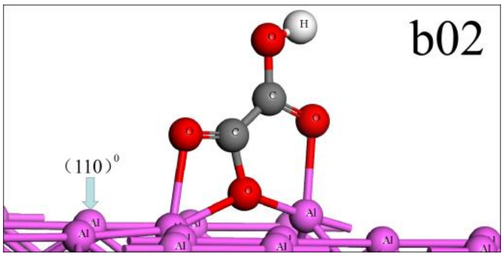

(b)02

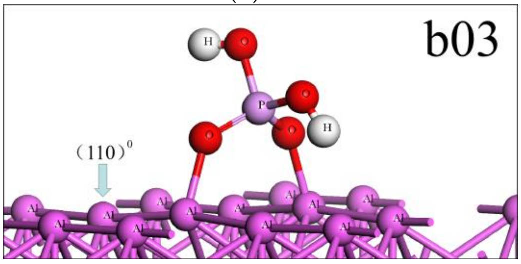

(b)03

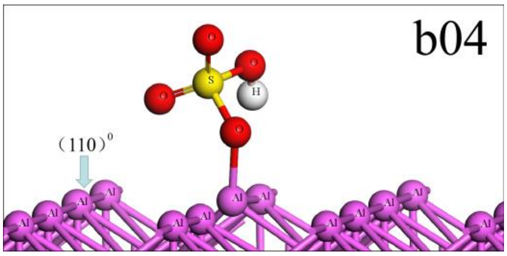

(b)04

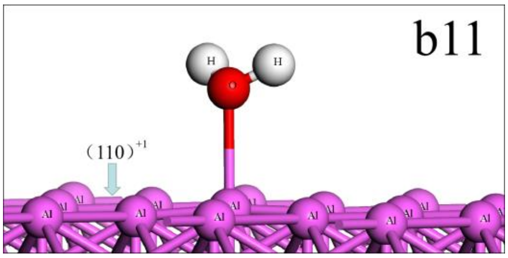

(b)11

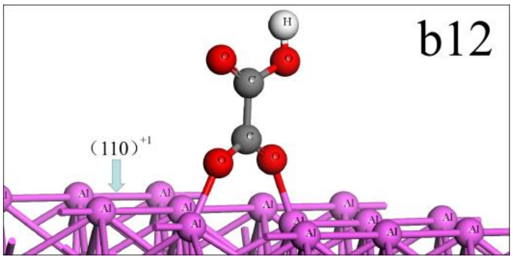

(b)12

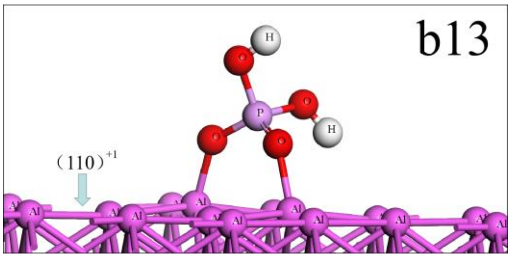

(b)13

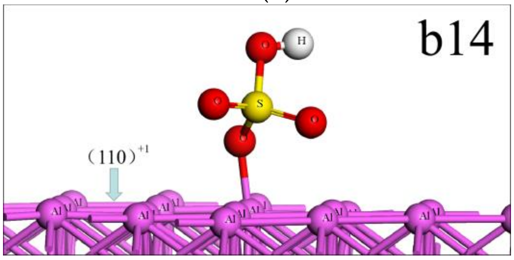

(b)14

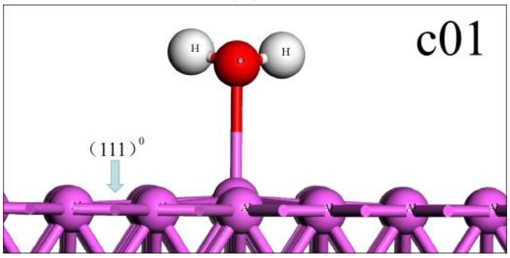

(c)01

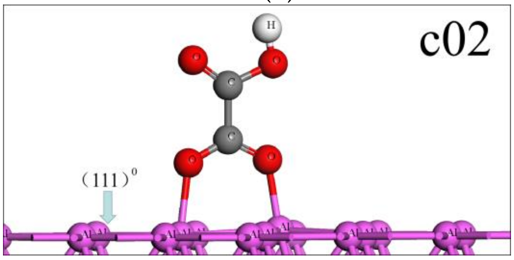

(c)02

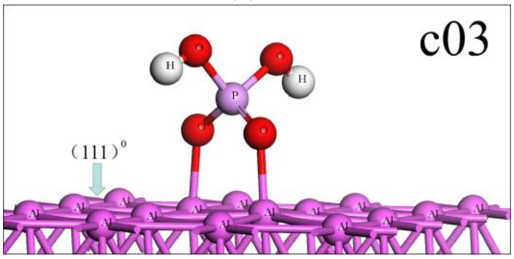

(c)03

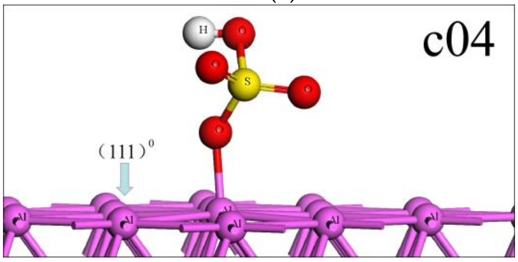

(c)04

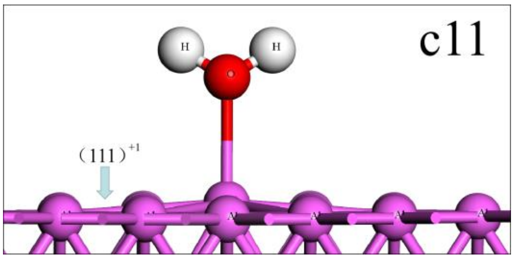

(c)11

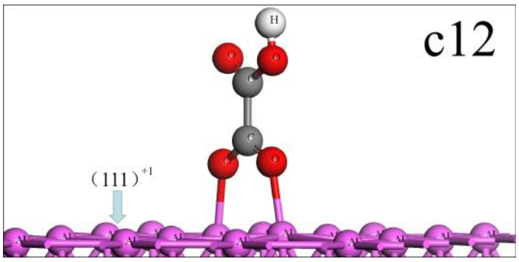

(c)12

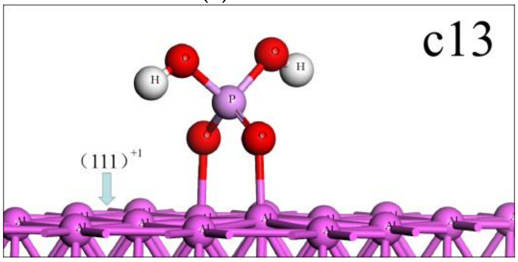

(c)13

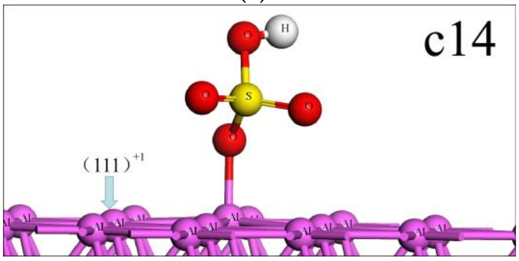

(c)14

Figure S2. The acid radicals and water adsorb on the natural and positively charged slabs. (a)01: the H_2O adsorbs on the natural (100) salb; (a)02: the $HC_2O_4^-$ adsorbs on the natural (100)

salb; (a)03: the $H_2PO_4^-$ adsorbs on the natural (100) salb; (a)04: the HSO_4^- adsorbs on the natural (100) salb; (a)11: the H_2O adsorbs on the positively charged (100) salb; (a)12: the $HC_2O_4^-$ adsorbs on the positively charged (100) salb; (a)13: the $H_2PO_4^-$ adsorbs on the positively charged (100) salb; (a)14: the HSO_4^- adsorbs on the positively charged (100) salb.

(b)01: the H_2O adsorbs on the natural (110) salb; (b)02: the $HC_2O_4^-$ adsorbs on the natural (110) salb; (b)03: the $H_2PO_4^-$ adsorbs on the natural (110) salb; (b)04: the HSO_4^- adsorbs on the natural (110) salb; (b)11: the H_2O adsorbs on the positively charged (110) salb; (b)12: the $HC_2O_4^-$ adsorbs on the positively charged (110) salb; (b)13: the $H_2PO_4^-$ adsorbs on the positively charged (110) salb; (b)14: the HSO_4^- adsorbs on the positively charged (110) salb.

(c)01: the H_2O adsorbs on the natural (111) salb; (c)02: the $HC_2O_4^-$ adsorbs on the natural (111) salb; (c)03: the $H_2PO_4^-$ adsorbs on the natural (111) salb; (c)04: the HSO_4^- adsorbs on the natural (111) salb; (c)11: the H_2O adsorbs on the positively charged (111) salb; (c)12: the $HC_2O_4^-$ adsorbs on the positively charged (111) salb; (c)13: the $H_2PO_4^-$ adsorbs on the positively charged (111) salb; (c)14: the HSO_4^- adsorbs on the positively charged (111) salb.

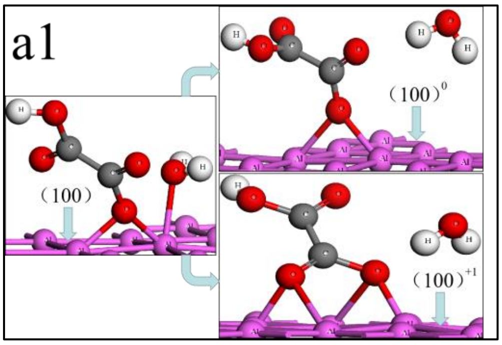

(a)1

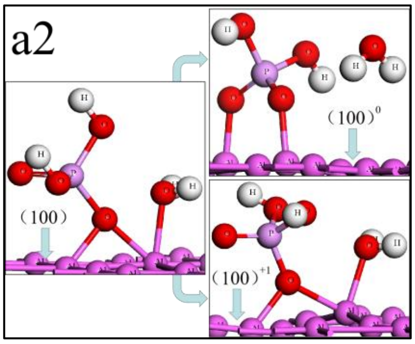

(a)2

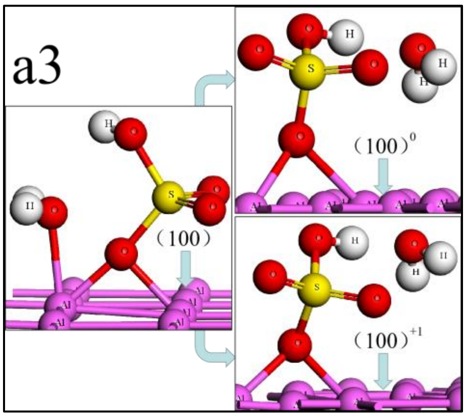

(a)3

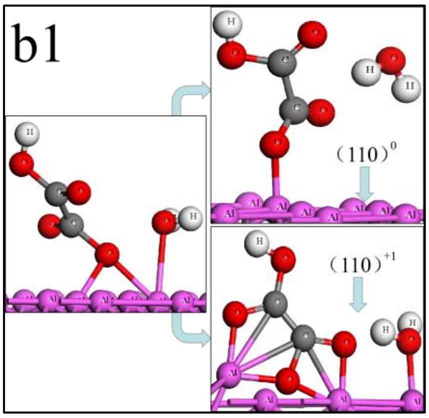

(b)1

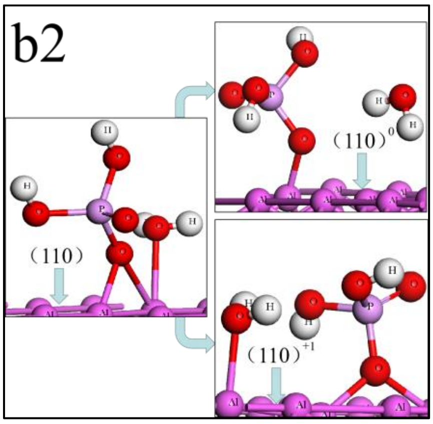

(b)2

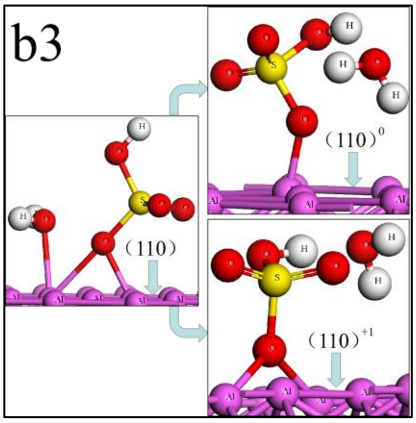

(b)3

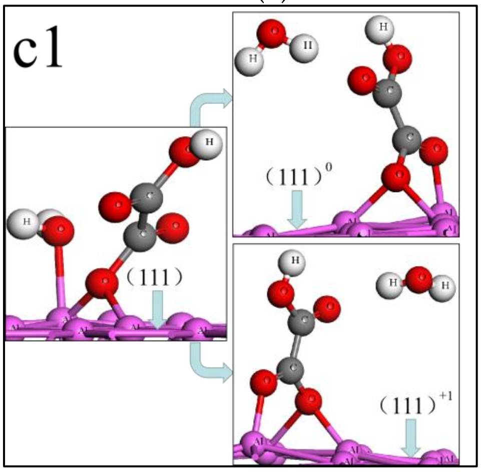

(c)1

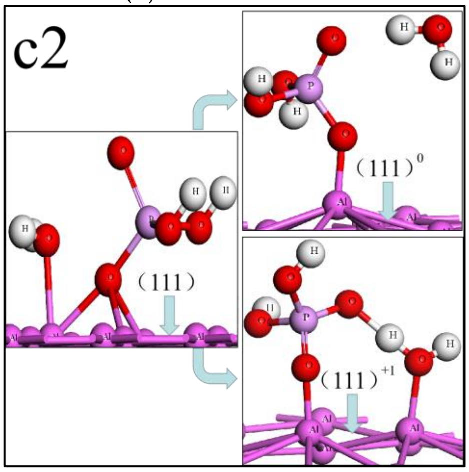

(c)2

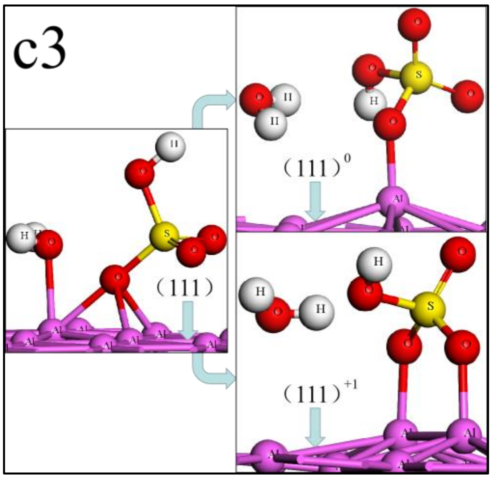

(c)3

Figure S3. The acid radicals substitute water on the natural and positively charged slabs.
(a)1: the $HC_2O_4^-$ substitute H_2O on the natural and positively charged slab of (100);
(a)2: the $H_2PO_4^-$ substitute H_2O on the natural and positively charged slab of (100);
(a)3: the HSO_4^- substitute H_2O on the natural and positively charged slab of (100);
(b)1: the $HC_2O_4^-$ substitute H_2O on the natural and positively charged slab of (110);
(b)2: the $H_2PO_4^-$ substitute H_2O on the natural and positively charged slab of (110);
(b)3: the HSO_4^- substitute H_2O on the natural and positively charged slab of (110);
(c)1: the $HC_2O_4^-$ substitute H_2O on the natural and positively charged slab of (111);
(c)2: the $H_2PO_4^-$ substitute H_2O on the natural and positively charged slab of (111);
(c)3: the HSO_4^- substitute H_2O on the natural and positively charged slab of (111);
